# Supplementary material for: Tunable self-healing of magnetically propelling colloidal carpets
Source: Nat Commun. 2019 Jun 4;10:2444. doi: 10.1038/s41467-019-10255-4 (PMC6547653; doi:10.1038/s41467-019-10255-4)
Supplement: Supplementary file 1 — Supplementary Information [file 41467_2019_10255_MOESM1_ESM.pdf]

Supplementary Information

Tunable self-healing of magnetically propelling colloidal carpets

Helena Massana-Cid[1] Fanlong Meng[2,3] Daiki Matsunaga,[4,3] Ramin Golestanian,[2, 3] Pietro Tierno[1, 5, 6]

[1]Departament de Física de la Matéria Condensada, Universitat de Barcelona, 08028 Barcelona, Spain

[2]Max Planck Institute for Dynamics and Self-Organization (MPIDS), 37077 Gottingen, Germany

[3]Rudolf Peierls Centre for Theoretical Physics, University of Oxford, Oxford OX1 3PU, United Kingdom

[4]Division of Bioengineering, Graduate School of Engineering Science, Osaka University, 5608531 Osaka, Japan

[5]Institut de Nanociéncia i Nanotecnologia, Universitat de Barcelona, Barcelona, Spain

[6]Universitat de Barcelona Institute of Complex Systems (UBICS), Universitat de Barcelona, Barcelona, Spain

Supplementary Figures

Supplementary Figure 1

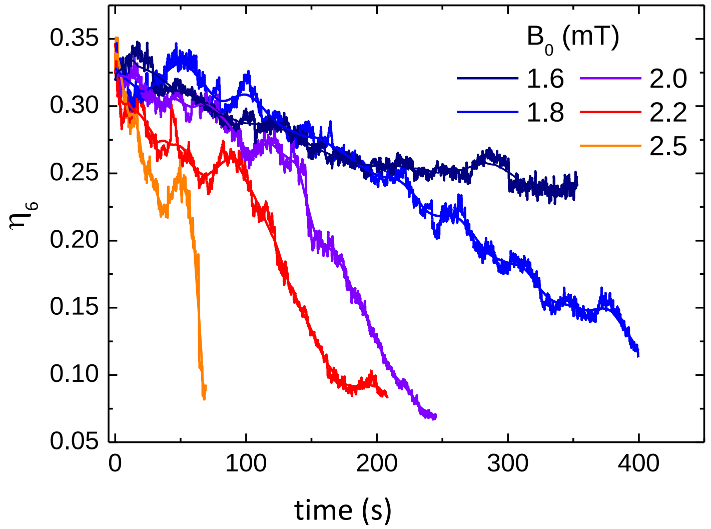

Caption Supplementary Figure 1. Exponent η_6 of the correlation function $g_6(r)$ versus time for different values of the magnetic field B_0, and hence carpet speed v_c.

Supplementary Figure 2

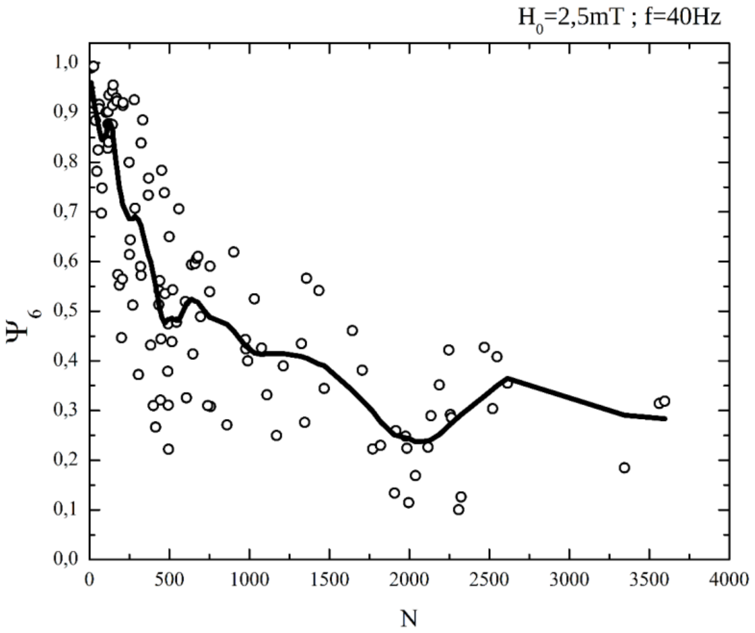

Caption Supplementary Figure 2. Bond orientational order parameter Ψ_6 versus the number of particles within the carpet in the assembly stage. The continuous black line is an average of the experimental data, which are the scattered open circles. The applied rotating field has amplitude B_0=*2.5 mT* and frequency *f = 40 Hz*.

Supplementary Figure 3

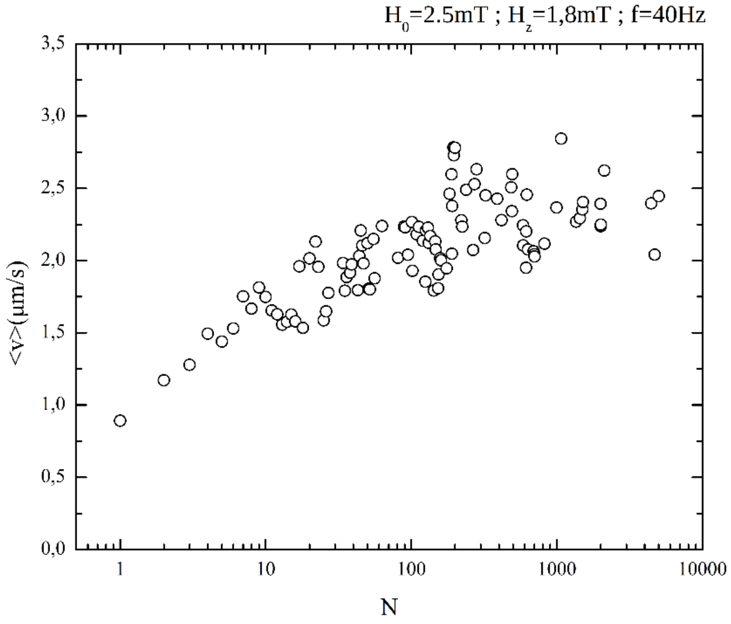

Caption Supplementary Figure 3. Average speed of the carpet versus the number of particles N for a carpet driven by a time dependent field with amplitudes B_0 *=2.5 mT,* B_z *=1.8 mT* and frequency *f = 40 Hz*.

Supplementary Figure 4

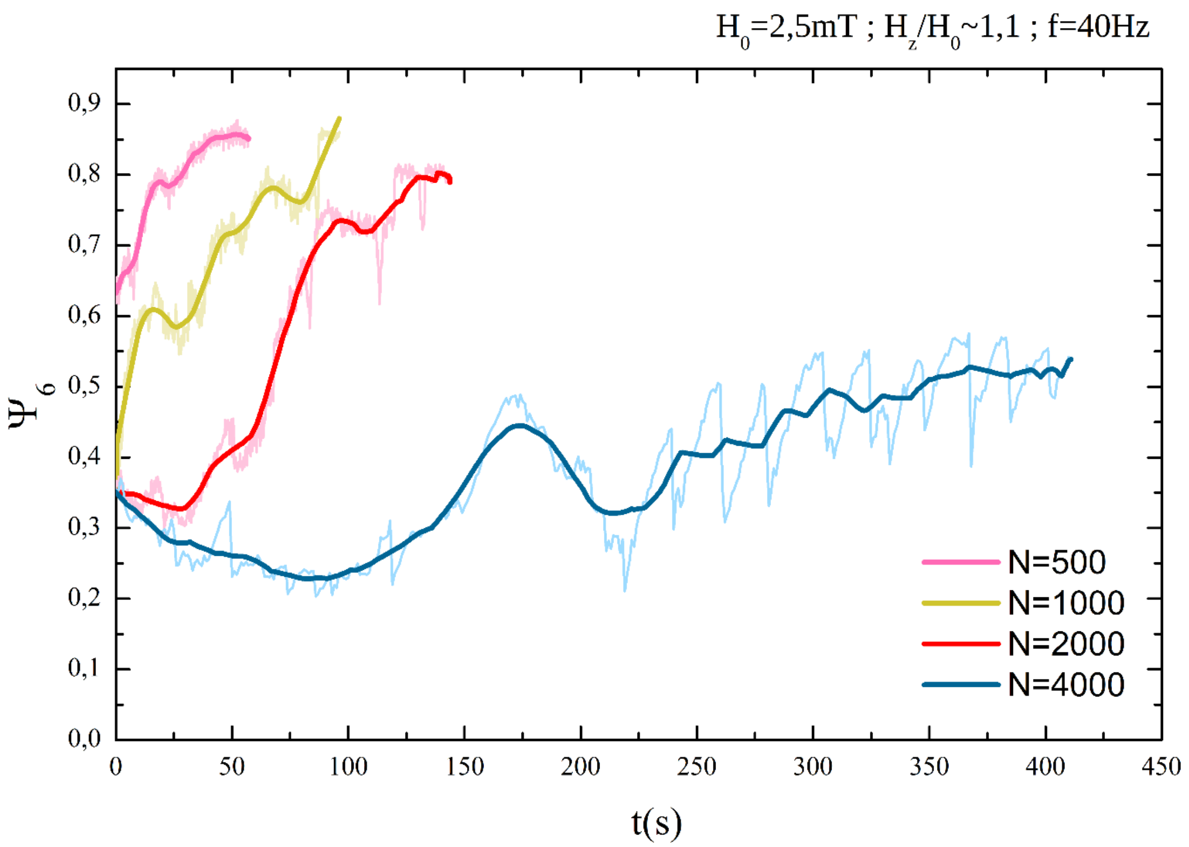

Caption Supplementary Figure 4. Bond orientational order parameter versus time for four different carpet sizes.
