# Supplementary material for: Tunable self-healing of magnetically propelling colloidal carpets
Source: Nat Commun. 2019 Jun 4;10:2444. doi: 10.1038/s41467-019-10255-4 (PMC6547653; doi:10.1038/s41467-019-10255-4)
Supplement: Supplementary file 3 — Description of Additional Supplementary Files [file 41467_2019_10255_MOESM3_ESM.pdf]

Description of Additional Supplementary Files

File Name: Supplementary Movie 1

Description: This videoclip illustrates the assembly of paramagnetic colloids in a two-dimensional carpet via a rotating magnetic field with amplitude B_0 = 1.6 mT and frequency f = 40 Hz. The carpet is later transported to the right via the application of a rotating field perpendicular to the plane of the particles with amplitudes B_0 = 1.6 mT and B_z = 1.7 mT, and frequency f = 40 Hz. The oscillating component applied along the *y*-axis has amplitude $B_y = B_0$ and frequency $f_y = f/2$. The video correspond to the Figs.1(d,e,f) of the article.

File Name: Supplementary Movie 2

Description: This videoclip illustrates the breakage of a two-dimensional carpet when the amplitude of the perpendicular component of the rotating field is raised above a limiting value; here B_z = 2.2 mT. The remaining field parameters are B_0 = 1.17 mT, f = 40 Hz and $f_y = f/2$. The video corresponds to the first of the two micrographs at the top of Fig.1(g) in the manuscript.

File Name: Supplementary Movie 3

Description: Generation of the colloidal flow above the carpet in the tread-milling regime. The video shows the motion of paramagnetic colloids above the carpet and was obtained by using the optical microscope in the reflection mode rather than the transmission one. The applied field parameters are B_0 = 2.2 mT, B_z = 1.8 mT, f = 40 Hz and $f_y = f/2$. The video corresponds to Fig.2(a) in the manuscript.

File Name: Supplementary Movie 4
Description: This video illustrates the three different possibilities that the particles propelling above the carpet have in the treadmilling phase: (i) filling one hole, (ii) traveling across the whole carpet, (iii) change direction due to a grain boundary. The applied field parameters are B_0 = 2 mT, B_z = 2 mT, f = 40 Hz and $f_y = f/2$. The video corresponds to the second of the two micrographs at the top of Fig.1(g).

File Name: Supplementary Movie 5

Description: Videos showing the transformation of a carpet composed by 1045 particles from a disordered phase with one grain boundary to a perfect crystalline lattice after 2 min of propulsion in the tread-milling phase. The applied field parameters are B_0 = 2.5 mT, B_z = 2.7 mT, f = 40 Hz and $f_y = f/2$. The video corresponds to the second of the two micrographs at the top of Fig.3(a) of the article.
